# Preventive Effects of Fermented Yak Milk-Derived *Lacticaseibacillus paracasei* CD12-1 Against DSS-Induced Colitis in Mice

**DOI:** 10.3390/foods15173120

**Published:** 2026-09-02

**Authors:** Hongqiang Li, Teng Zhen, Junyang Li, Furong Han, Xian Guo, Defu Tang, Cheng Peng

**Affiliations:** 1 College of Animal Science and Technology, Gansu Agricultural University, Lanzhou 730070, China; lhq136531597@163.com (H.L.); 1073323010045@st.gsau.edu.cn (T.Z.); ljy2413197980@outlook.com (J.L.); 2 Gansu Animal Husbandry Technology Extension Station, Lanzhou 730030, China; nbx1208@163.com; 3 Key Laboratory of Animal Genetics and Breeding on Tibetan Plateau, Ministry of Agriculture and Rural Affairs, Key Laboratory of Yak Breeding Engineering of Gansu Province, Lanzhou Institute of Husbandry and Pharmaceutical Sciences, Chinese Academy of Agricultural Sciences, Lanzhou 730050, China; guoxian@caas.cn

**Keywords:** *Lacticaseibacillus paracasei* CD12-1, ulcerative colitis, intestinal barrier, gut microbiota, DSS-induced colitis

## Abstract

Ulcerative colitis is a chronic inflammatory bowel disease characterized by mucosal barrier disruption, dysregulated immune responses, and gut microbial imbalance. However, limitations of current therapies highlight the need for safe probiotic interventions. This study evaluated the preventive effects of fermented yak milk-derived *Lacticaseibacillus paracasei* CD12-1 on dextran sulfate sodium (DSS)-induced colitis in mice. The results showed that DSS caused body weight loss, an increased disease activity index, colon shortening, and severe histopathological injury. CD12-1 alleviated these abnormalities with differential effects across doses. The low-dose treatment produced the most comprehensive improvements in histopathological damage, goblet cell abundance, tight junction integrity, inflammatory cytokines, and short-chain fatty acids, whereas the high dose more effectively attenuated body weight loss. CD12-1 increased colonic ZO-1 and Occludin expression, reduced IL-1β, IL-6, and TNF-α levels, and increased IL-10. The low dose also elevated acetate and butyrate levels. Gut microbiota analysis showed that CD12-1 was associated with changes in the relative abundances of *Lactobacillus*, *Bifidobacterium*, *Allobaculum*, *Akkermansia*, and several inflammation-associated taxa. Correlation analysis associated *Lactobacillus* and *Allobaculum* with milder disease and improved barrier-related indicators, whereas *Bacteroides* and *Sutterella* were associated with greater disease severity and inflammation. Overall, CD12-1 alleviated DSS-induced colitis by improving intestinal barrier integrity and inflammatory homeostasis, accompanied by changes in microbial composition and short-chain fatty acid production.

## 1. Introduction

Ulcerative colitis (UC) is a chronic, relapsing inflammatory bowel disease that primarily affects the colonic and rectal mucosa [1,2]. Its development and progression are closely associated with multiple factors, including gut microbial dysbiosis, disruption of the mucosal barrier, and dysregulation of immune homeostasis [3]. Although current pharmacological therapies can induce and maintain remission, some patients experience limited efficacy, adverse effects associated with long-term medication, and disease recurrence after treatment withdrawal [4,5,6]. Moreover, impaired gut microbial homeostasis and mucosal barrier function may not be fully restored [7]. Therefore, developing safe and effective microbiota-targeted strategies suitable for long-term adjunctive intervention is of considerable importance for the prevention and management of UC.

Probiotics and probiotic-derived products have received increasing attention because of their potential to modulate the intestinal ecosystem and improve host health [8]. Certain lactic acid bacteria can alleviate intestinal inflammation and preserve mucosal barrier function by regulating gut microbial composition and metabolic activity, maintaining the mucus layer and tight junction integrity, suppressing excessive inflammatory responses, and modulating mucosal immune homeostasis [1,9,10]. However, probiotic effects are highly strain-specific, and functional properties identified through in vitro assays or genomic prediction do not necessarily reflect efficacy within the complex host environment [11,12]. Accordingly, the anti-inflammatory and barrier-protective effects of candidate probiotic strains require further validation in well-designed animal studies.

In our previous work, *Lacticaseibacillus paracasei* CD12-1, a strain with promising probiotic potential, was isolated from traditional fermented yak milk [13]. The strain exhibited strong tolerance to acidic conditions, bile salts, and simulated gastrointestinal environments, together with antioxidant activity and a favorable safety profile. Genomic analysis further revealed multiple candidate genes potentially associated with environmental adaptation and probiotic functions. These characteristics suggested that CD12-1 could survive gastrointestinal transit and retain functional activity in the intestinal environment, providing a biological rationale for evaluating its potential against intestinal inflammatory injury. However, whether CD12-1 could protect against colitis and modulate intestinal barrier function and gut microbial homeostasis had not been investigated. Therefore, the present study employed a dextran sulfate sodium (DSS)-induced mouse model of experimental colitis to systematically evaluate the preventive effects of different doses of CD12-1. Disease activity, colon length, histopathological injury, goblet cell abundance, tight junction protein expression, inflammatory cytokine levels, short-chain fatty acid concentrations, and gut microbial alterations were assessed to explore the potential pathways through which this strain alleviates colitis and to provide an experimental basis for its further development as a candidate probiotic.

## 2. Materials and Methods

### 2.1. Preparation of CD12-1 Strain

CD12-1 was activated and subcultured, harvested by centrifugation at 4500 rpm for 10 min, and washed three times with sterile phosphate-buffered saline (PBS; Biosharp, Beijing Labgic Technology Co., Ltd., Beijing, China). Based on the previous study [10], the bacterial suspensions were adjusted to 1 × 10^8^, 1 × 10^9^, and 1 × 10^10^ CFU/mL. Fresh suspensions were prepared daily throughout the experiment and administered immediately by oral gavage using disposable sterile syringes.

### 2.2. Animal Experiments

Sixty 6-week-old specific pathogen-free (SPF) female C57BL/6 mice (18 ± 2 g) were obtained from the Laboratory Animal Center of the Lanzhou Veterinary Research Institute, Chinese Academy of Agricultural Sciences (Gansu, China). Mice were housed at 23 ± 2 °C and 50 ± 5% relative humidity under a 12 h light/dark cycle. Sterile bedding was replaced every 2 days, and food and water were provided ad libitum. Before the experiment, the mice were acclimatized for 1 week. The animal trial protocol was approved by the Ethics Committee of Laboratory Animal Management and Animal Welfare of Gansu Agricultural University (No. GSAU-Eth-VMC-2022-016, approval date: 10 March 2022).

After acclimation, the mice were randomly assigned to five groups (n = 12 per group): normal control (C), DSS model (D), low-dose (L), medium-dose (M), and high-dose (H) groups. Within each group, six mice were randomly selected based on individual animal IDs as the biological analysis cohort, without reference to body weight, disease activity index (DAI), or any other experimental outcome. The same six mice were consistently used for all subsequent phenotypic, histological, biochemical, short-chain fatty acids (SCFAs), and gut microbiota analyses to ensure individual-level matching across endpoints.

**Figure 1:**
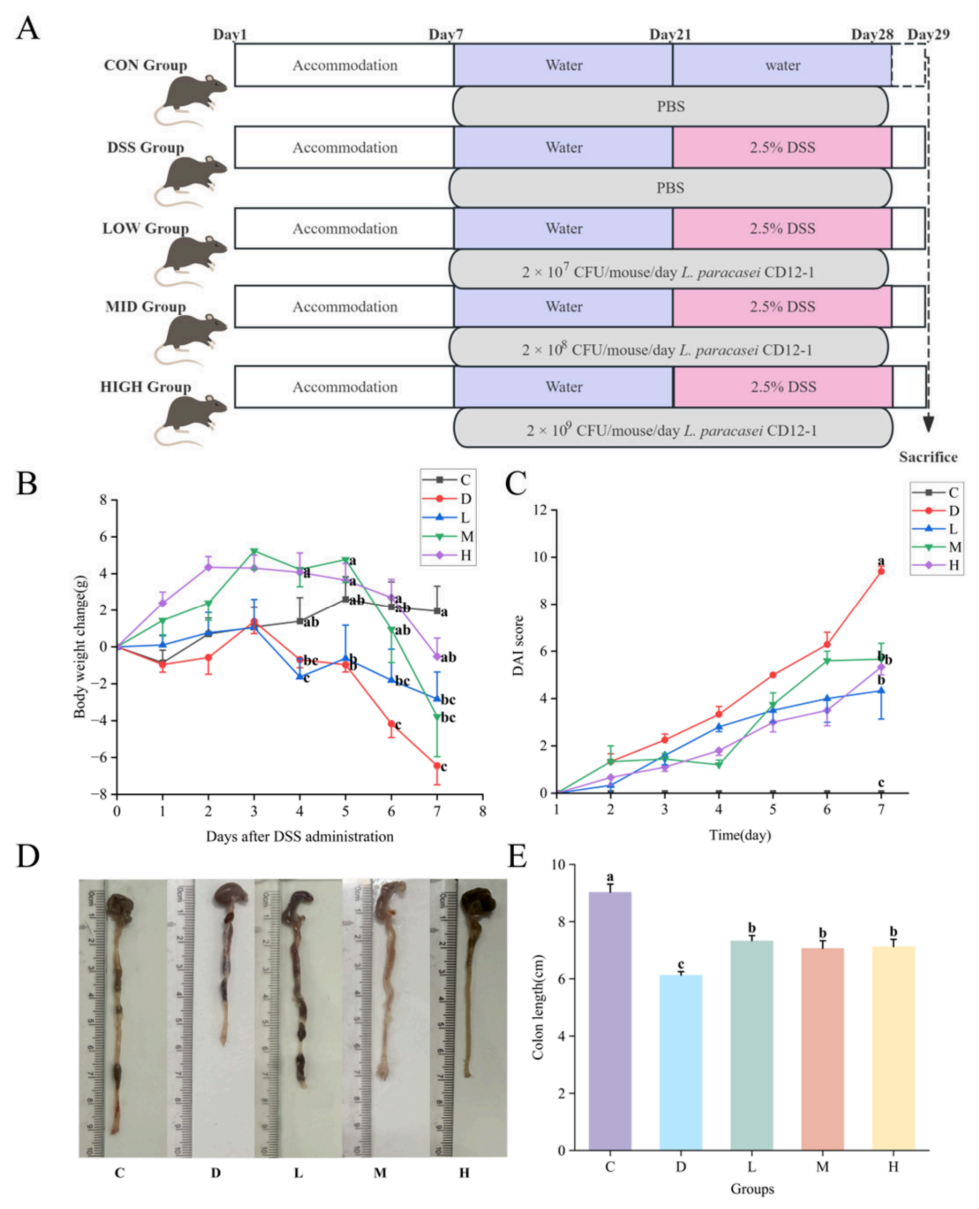
CD12-1 improved the symptoms of colitis. (**A**) Mice model of colitis induced by 2.5% DSS; (**B**) changes in body weight of mice after DSS administration; (**C**) DAI score of colitis in mice; (**D**) Representative macroscopic pictures of the colon; (**E**) colon length. Data are presented as mean ± SEM (n = 6 mice per group). Different letters indicate significant differences among groups (*p* < 0.05).

The experiment lasted 3 weeks. Mice in the C and D groups received 0.2 mL of sterile PBS daily by oral gavage, whereas those in the L, M, and H groups received 0.2 mL of CD12-1 suspensions at 1 × 10^8^, 1 × 10^9^, and 1 × 10^10^ CFU/mL, corresponding to 2 × 10^7^, 2 × 10^8^, and 2 × 10^9^ CFU/mouse/day, respectively. During the first 2 weeks, all groups had free access to sterile distilled water. During the third week, mice in all groups except the C group were provided 2.5% (*w*/*v*) DSS (MW: 36,000–50,000 Da; MP Biomedicals, LLC, Solon, OH, USA) in drinking water ad libitum to induce colitis. Oral gavage was continued throughout the 3-week experimental period. The experimental design is illustrated in Figure 1A. At the end of the experiment, the mice were fasted for 12 h and euthanized by cervical dislocation. The entire colon was excised, measured for length, and rinsed with sterile PBS. A portion of the colon was fixed in 4% paraformaldehyde (Wuhan Servicebio Technology Co., Ltd., Wuhan, China) for subsequent histological analysis. The remaining colonic tissues and cecal contents were immediately snap-frozen in liquid nitrogen and stored at −80 °C until further analysis.

### 2.3. Assessment of Body Weight, Colon Length, and Disease Activity Index

Food intake and body weight were recorded daily throughout the experiment. Stool consistency and fecal bleeding were also assessed daily, and the DAI was calculated according to the scoring criteria described by Ma et al. [14], as detailed in Table 1. At necropsy, the entire colon was excised, and its length was measured without stretching.

### 2.4. Colonic Histological Examination and Histopathological Scoring

Following collection, a segment of the distal colon approximately 1 cm proximal to the anus was excised and gently cleared of luminal contents. The tissue was fixed in 4% paraformaldehyde, paraffin-embedded, sectioned, and stained with hematoxylin and eosin (H&E). Histopathological damage was scored according to the criteria described by Cooper et al. [15].

### 2.5. Periodic Acid–Schiff Staining of Colonic Goblet Cells

Colon tissues were fixed overnight in 4% paraformaldehyde, routinely processed, paraffin-embedded, and sectioned at 5 μm. The sections were stained with periodic acid–Schiff (PAS), examined under a light microscope, and imaged for subsequent analysis.

### 2.6. Immunohistochemical Analysis of Colonic Tissues

Paraffin-embedded colon tissues were sectioned at 5 μm, deparaffinized in xylene twice for 15 min each, and rehydrated through graded ethanol solutions. Antigen retrieval was performed in citrate antigen-retrieval buffer (pH 6.0) using microwave heating for 8 min, followed by an 8 min interval and an additional 7 min of heating. After natural cooling to room temperature, sections were washed three times with PBS (pH 7.4) for 5 min each. Endogenous peroxidase activity was blocked with 3% H_2_O_2_ at room temperature in the dark for 25 min. After washing, nonspecific binding was blocked with 3% bovine serum albumin (BSA) at room temperature for 30 min. Sections were incubated overnight at 4 °C in a humidified chamber with primary antibodies against ZO-1 (Sanying; Wuhan, China; catalogue no. 21773-1-AP; dilution 1:100), Occludin (Sanying; Wuhan, China; catalogue no. 27260-1-AP; dilution 1:100), and Claudin-1 (Affinity Biosciences; Liyang, China; catalogue no. BF3237; dilution 1:200). After three washes with PBS for 5 min each, the sections were incubated with an HRP-conjugated goat anti-rabbit secondary antibody (SeraCare; Milford, CT, USA; catalogue no. 5220-0336; dilution 1:500) at room temperature for 50 min in the dark. Immunoreactivity was visualized using 3,3′-diaminobenzidine (DAB) for 5 min, followed by hematoxylin counterstaining. The sections were differentiated briefly in 1% hydrochloric acid alcohol, washed, dehydrated through graded ethanol, cleared in xylene, and mounted with neutral resin. Immunohistochemically stained sections were imaged using CaseViewer software version 2.4. Images were quantitatively analyzed using ImageJ version 1.54p, and the average optical density (AOD) of positively stained regions was calculated.

### 2.7. Measurement of Inflammatory Cytokines in Colonic Tissues

The concentrations of interleukin-1β (IL-1β), IL-6, IL-10, and tumor necrosis factor-α (TNF-α) in colonic tissues were measured using enzyme-linked immunosorbent assay (ELISA) kits (Elabscience Biotechnology Co., Ltd., Wuhan, China) according to the manufacturers’ instructions.

### 2.8. Intestinal Microbial Analysis

Cecal content samples from the same six mice described in Section 2.2 were used for 16S rRNA gene sequencing. Microbial DNA was extracted from intestinal content samples using the MagBeads FastDNA Kit for Soil (MP Biomedicals, Irvine, CA, USA) according to the manufacturer’s instructions. DNA integrity was assessed by 0.8% agarose gel electrophoresis, and DNA concentration and purity were determined using a NanoDrop spectrophotometer. The V3–V4 region of the bacterial 16S rRNA gene was amplified using the primers 338F (5′-barcode-ACTCCTACGGGAGGCAGCA-3′) and 806R (5′-GGACTACHVGGGTWTCTAAT-3′). PCR products were pooled in equimolar amounts based on their concentrations and subjected to Illumina NovaSeq 6000 platform sequencing.

Raw sequencing reads were demultiplexed using the QIIME 2 demux plugin, and primer sequences were removed using the cutadapt plugin. Quality filtering, denoising, paired-end read merging, and chimera removal were performed using the DADA2 plugin. Amplicon sequence variants (ASVs) and their corresponding feature-abundance table were generated directly from the denoised sequences. Downstream microbiome analyses were conducted using QIIME 2 version 2024.5 following the official workflow with dataset-specific parameter settings.

### 2.9. Determination of SCFAs

In total, 20 mg of gut content was transferred to a 2 mL centrifuge tube containing two stainless-steel beads and extracted with 800 μL of extraction solvent containing an internal standard. After vortexing for 60 s, the sample was homogenized twice at 55 Hz for 60 s each and centrifuged at 4000× *g* and 10 °C for 10 min. An aliquot of 40 μL of the supernatant was sequentially mixed with 20 μL of 200 mM 3-nitrophenylhydrazine (3-NPH) and 20 μL of 120 mM 1-ethyl-3-(3-dimethylaminopropyl) carbodiimide hydrochloride (EDC·HCl) containing 6% pyridine. Derivatization was performed at 40 °C with shaking at 1200 rpm for 30 min. After cooling on ice for 3 min, the reaction mixture was centrifuged at 12,000× *g* and 4 °C for 10 min. Subsequently, 50 μL of the supernatant was diluted with 150 μL of water containing 0.1% (*v*/*v*) formic acid, vortexed for 60 s, and centrifuged again under the same conditions. The final supernatant was filtered through a 0.22 μm membrane, transferred to an autosampler vial, and analyzed for SCFAs by liquid chromatography–tandem mass spectrometry (LC–MS/MS; SCIEX, Framingham, MA, USA).

### 2.10. Statistical Analysis

Data were organized using Microsoft Excel 2021 and statistically analyzed using IBM SPSS Statistics version 27.0 (IBM Corp., Armonk, NY, USA). All analyses were based on six biologically independent mice per group, and data are presented as mean ± standard error of the mean (SEM). For continuous endpoint variables, normality was assessed using the Shapiro–Wilk test and homogeneity of variances using Levene’s test. Data satisfying the assumptions of normality and homogeneity of variance were analyzed by one-way analysis of variance (ANOVA), followed by Duncan’s multiple range test for post hoc comparisons. When the assumption of homogeneity of variance was violated, Welch’s ANOVA followed by the Games–Howell test was used. Non-normally distributed data were analyzed using the Kruskal–Wallis test followed by Dunn’s multiple-comparison test. Body weight and DAI were analyzed using two-way repeated-measures ANOVA followed by Sidak’s multiple comparisons test. Differences were considered statistically significant at *p* < 0.05.

## 3. Results

### 3.1. CD12-1 Improved the Symptoms of UC

Changes in body weight over time of mice during the 7-day DSS induction period are shown in Figure 1B. During model establishment, mice in the C group exhibited a continuous but gradual increase in body weight, remained in good overall health, and showed a normal growth pattern. In contrast, the body weight of mice in the D group began to decrease markedly from day 3, with the greatest reduction observed on day 7, consistent with the typical pathological manifestations of DSS-induced colitis, including anorexia and dehydration. Compared with the D group, all three intervention groups exhibited varying degrees of protection against DSS-induced body weight loss.

Figure 1C shows the dynamic changes in DAI scores during DSS-induced colitis. The DAI score of the C group remained consistently low throughout the experimental period, with no apparent changes. In contrast, the DAI score of the D group progressively increased following DSS administration and reached its highest level on day 7, when it was significantly higher than that of the C group (*p* < 0.05), confirming the successful establishment of the colitis model. Compared with the D group, the L, M, and H groups all exhibited varying degrees of attenuation in the DSS-induced increase in DAI. On day 7, DAI scores in all three intervention groups were significantly lower than that in the D group (*p* < 0.05), although they remained higher than that in the C group.

Mice in the C group had the longest colons, with a mean length of 8.83 ± 0.32 cm. Compared with the C group, DSS administration significantly shortened the colon, with the mean colon length decreasing to 6.13 ± 0.12 cm in the D group. Following intervention with CD12-1, the mean colon lengths in the L, M, and H groups were 7.02 ± 0.21, 7.07 ± 0.26, and 7.02 ± 0.25 cm, respectively, all of which were greater than that observed in the D group (Figure 1D). Statistical analysis showed that colon length was significantly increased in the L, M, and H groups compared with the D group (*p* < 0.05), whereas no significant differences were detected among the three dose groups (*p* > 0.05, Figure 1E).

### 3.2. CD12-1 Administration Reduced Histological Injury and Goblet Cell Depletion

**Figure 2:**
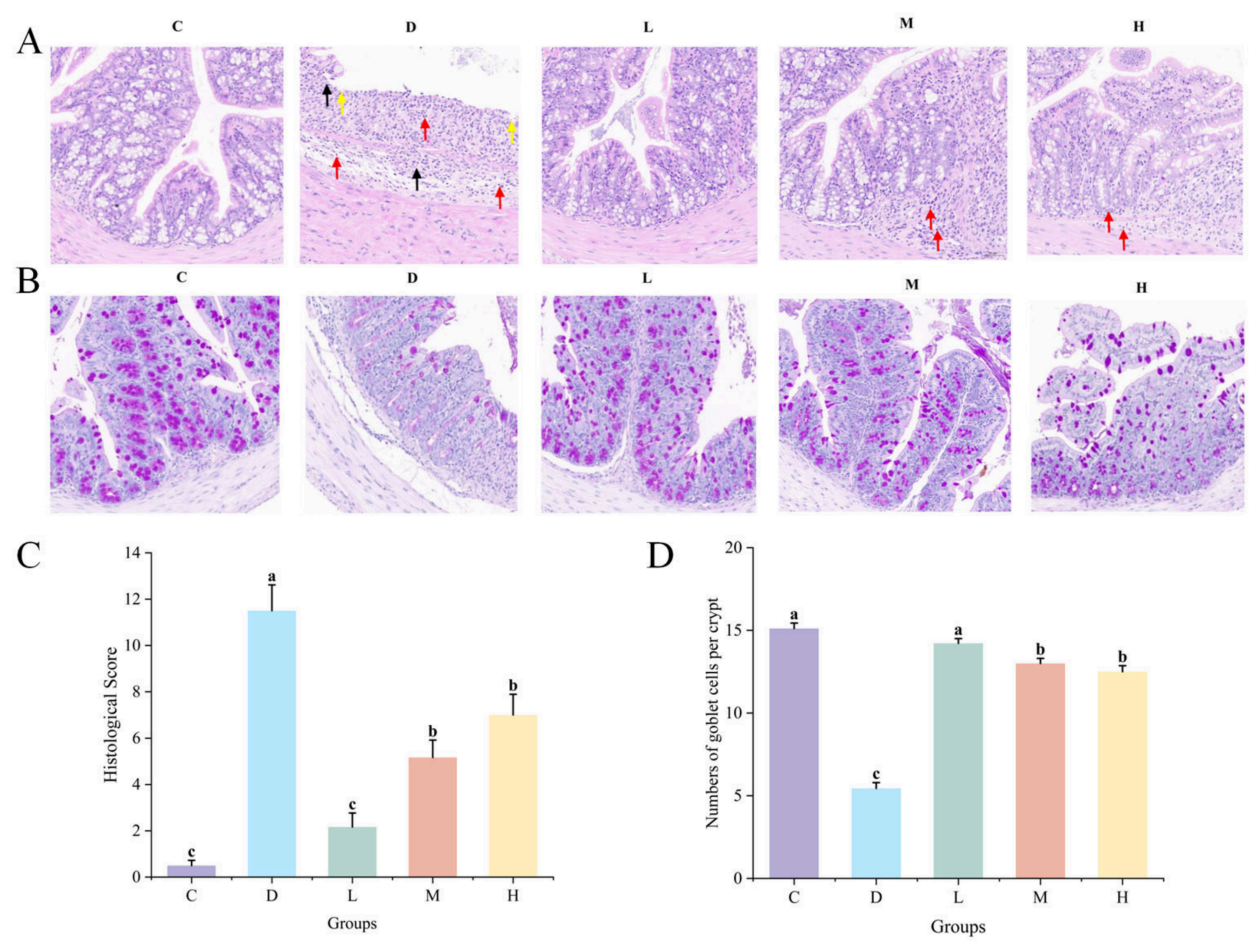
CD12-1 administration mitigated the histopathological colon injury and goblet cell loss. (**A**) Representative pictures of H&E-stained colon tissue and (**C**) histological score of colitis in mice; (**B**) representative pictures of PAS-stained colon tissues and (**D**) number of goblet cells per unit length (piece/mm). Data are presented as mean ± SEM (n = 6 mice per group). Different letters indicate significant differences among groups (*p* < 0.05).

Colonic histomorphology was evaluated by H&E staining (Figure 2A). Mice in the C group exhibited normal colonic architecture. In contrast, the D group showed severe histopathological damage, characterized by mucosal epithelial sloughing and exposure of the lamina propria (yellow arrows), cellular necrosis with nuclear pyknosis and fragmentation (black arrows), crypt atrophy and loss, and pronounced inflammatory cell infiltration (red arrows). Accordingly, the histopathological score was significantly higher than that of the C group (*p* < 0.05, Figure 2C). The M and H groups showed comparable histopathological improvements, including partial restoration of the mucosal structure and increased crypt numbers, although crypt organization remained irregular, with persistent inflammatory cell infiltration (red arrows) and focal mucosal edema. In the L group, low-dose CD12-1 treatment largely preserved normal colonic architecture, as evidenced by an intact and well-organized epithelium, minimal epithelial shedding or necrosis, abundant and clearly defined crypts, and limited inflammatory cell infiltration. Treatment with CD12-1 significantly reduced histopathological scores compared with the D group (*p* < 0.05), while no significant difference was observed between the L and C groups (*p* > 0.05).

Figure 2B shows the PAS staining of colonic tissues from each group. The C group exhibited intact crypt architecture, abundant goblet cells throughout the epithelium, and well-preserved cellular morphology, indicating sufficient mucin production and an intact mucosal barrier. In contrast, the D group showed a significant reduction in goblet cell numbers compared with the C group (*p* < 0.05), accompanied by crypt disruption, epithelial thinning, reduced goblet cell size, weakened PAS staining, and focal mucosal damage. These findings indicated that DSS impaired the colonic mucus layer and goblet cell function. Treatment with CD12-1 markedly alleviated DSS-induced goblet cell damage. Compared with the D group, all dose groups showed significantly less goblet cell loss (*p* < 0.05, Figure 2D). Notably, goblet cell numbers in the L group did not differ significantly from those in the C group (*p* > 0.05).

### 3.3. CD12-1 Administration Improved Intestinal Epithelial Barrier Functions in DSS-Induced Mice

**Figure 3:**
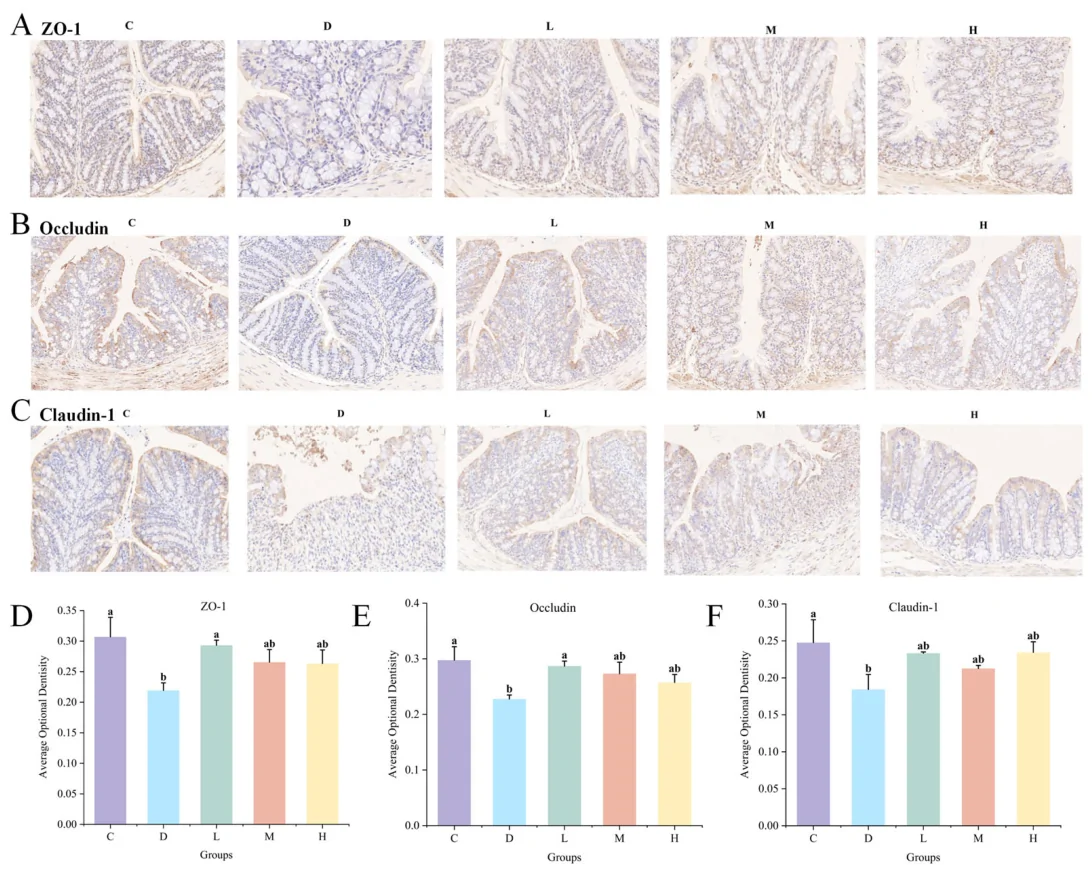
Effects of CD12-1 on intestinal epithelial barrier functions in DSS-induced mice. Representative pictures of ZO-1 (**A**), Occludin (**B**), and Claudin-1 (**C**) with IHC-staining in colon tissues and ZO-1 (**D**), Occludin (**E**), and Claudin-1 (**F**) average optical density in per group. Data are presented as mean ± SEM (n = 6 mice per group). Different letters indicate significant differences among groups (*p* < 0.05).

The expression and distribution of the tight junction proteins ZO-1, Occludin, and Claudin-1 in mouse colonic tissues were evaluated by IHC staining. In the C group, ZO-1 was highly and uniformly expressed between colonic epithelial cells, forming a continuous honeycomb-like pattern with intense staining (Figure 3A). DSS treatment markedly disrupted this pattern in the D group, as evidenced by reduced ZO-1 AOD and weak, discontinuous staining. Following CD12-1 treatment, ZO-1 AOD increased in all intervention groups. The increase was significant in the L group compared with the D group (*p* < 0.05), whereas no significant difference was observed between the L and C groups (*p* > 0.05, Figure 3D). Compared with the C group, the D group showed significantly reduced Occludin AOD in colonic tissues, accompanied by markedly weaker staining (Figure 3B,E). Low-dose CD12-1 treatment significantly increased Occludin AOD and enhanced its relatively uniform distribution within the intestinal glands, with no significant difference from the C group (*p* > 0.05). Although Occludin AOD tended to increase in the M and H groups relative to the D group, the differences were not significant (*p* > 0.05), and the improvement was less pronounced than that observed in the L group. Claudin-1 AOD were significantly lower in the D group than in the C group (*p* < 0.05, Figure 3C,F). Following CD12-1 treatment, Claudin-1 AOD showed an increasing trend, although the differences from the D group were not significant (*p* > 0.05). Claudin-1 expression also did not differ significantly among the three dose groups (*p* > 0.05).

### 3.4. CD12-1 Administration Mitigated Inflammatory Cytokine Levels in Colon Tissues

**Figure 4:**
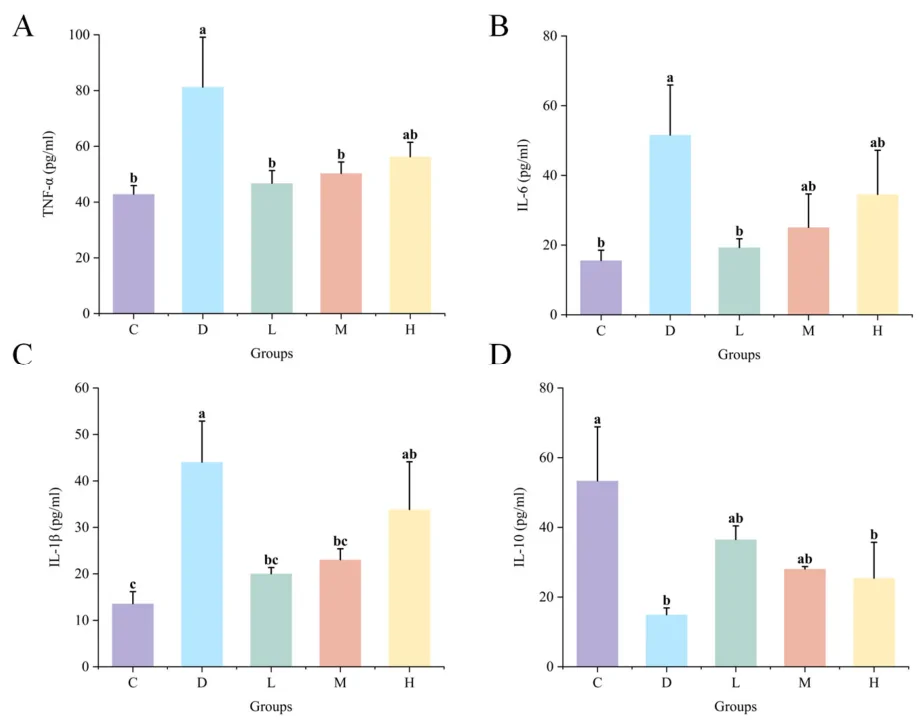
Effects of CD12-1 administration on inflammatory cytokines secretion. (**A**) TNF-α, (**B**) IL-6, (**C**) IL-1β, and (**D**) IL-10 level in colon. Data are presented as mean ± SEM (n = 6 mice per group). Different letters indicate significant differences among groups (*p* < 0.05).

Figure 4 shows the levels of colonic inflammatory cytokines in each group. The pro-inflammatory cytokines TNF-α, IL-6, and IL-1β were significantly elevated in the D group compared with the C group (*p* < 0.05, Figure 4A–C), whereas all three showed decreasing trends following CD12-1 treatment. TNF-α and IL-1β levels were significantly lower in the L and M groups than in the D group and approached normal levels (*p* < 0.05). IL-6 was also significantly reduced in the L group (*p* < 0.05), with no significant difference from the C group (*p* > 0.05). Although all three cytokines tended to decrease in the H group, the differences from the D group were not significant (*p* > 0.05).

Among the anti-inflammatory cytokines, IL-10 was highest in the C group (Figure 4D). DSS treatment significantly reduced IL-10 levels in the D group compared with the C group (*p* < 0.05), indicating suppression of the anti-inflammatory response during colitis. Following CD12-1 treatment, IL-10 levels were numerically increased in the L and M groups, with a greater recovery in the L group; however, neither group differed significantly from the C or D group (*p* > 0.05). In contrast, IL-10 remained low in the H group and was comparable to that in the D group.

### 3.5. CD12-1 Regulated the Gut Microbiota Ruined by DSS

**Figure 5:**
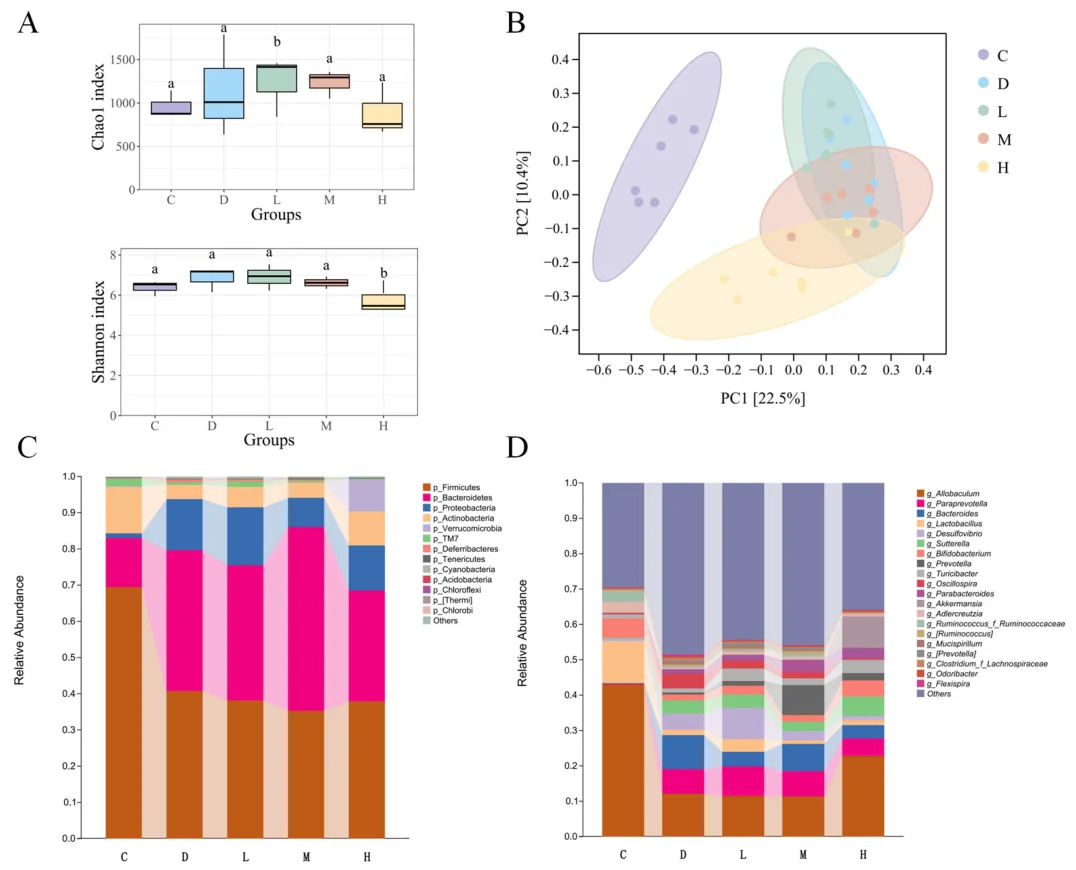
CD12-1 modulated the gut microbiota structure. (**A**) Chao1 and Shannon index; (**B**) PCoA plot with Bray–Curtis distance; average relative abundances of gut microbiota at the phylum (**C**) and genus (**D**) levels. Data are presented as mean ± SEM (n = 6 mice per group). Different letters indicate significant differences among groups (*p* < 0.05).

To explore the effect of CD12-1 on DSS-stimulated colitic mice, the sequencing of 16S rRNA was analyzed to study the changes in microbial community diversity. Alpha diversity analysis showed that the low-dose CD12-1 group had a significantly higher Chao1 index than the other groups, whereas the Shannon index was significantly lower in the high-dose group (*p* < 0.05, Figure 5A). Bray–Curtis-based PCoA revealed a clear separation between the C group and all DSS-exposed groups, while the D, L, and M groups largely overlapped (Figure 5B). The H group displayed a relatively distinct distribution. Collectively, these results indicate that CD12-1 differentially modulated gut microbial diversity and community structure across the tested doses.

The relative abundance of bacterial taxa at different taxonomic levels was determined to analyze the composition of the gut microbial community. At the phylum level, Bacteroidetes, Firmicutes, Actinobacteria, and Proteobacteria were the predominant phyla across all groups (Figure 5C). As shown in Figure S1A, compared with the C group, the D group exhibited significantly lower relative abundances of Firmicutes and Actinobacteria but significantly higher relative abundances of Bacteroidetes and Proteobacteria (*p* < 0.05). Following CD12-1 treatment, the relative abundance of Actinobacteria showed an increasing trend, with a significantly higher relative abundance in the H group than in the D group (*p* < 0.05). The relative abundance of Bacteroidetes remained significantly higher than that in the C group (*p* < 0.05) but did not differ significantly from that in the D group (*p* > 0.05). Firmicutes abundance was comparable among the D, L, M, and H groups. At the genus level (Figure 5D and Figure S1B), compared with the C group, the D group showed significantly lower relative abundances of *Allobaculum*, *Lactobacillus*, *Bifidobacterium*, and *Ruminococcus*, but significantly higher relative abundances of *Bacteroides* and *Oscillibacter* (*p* < 0.05). Following CD12-1 treatment, the relative abundance of *Allobaculum* tended to increase and was significantly higher in the H group than in the D group (*p* < 0.05). Conversely, *Oscillibacter* tended to decrease and was significantly less abundant in the H group than in the D group (*p* < 0.05). No significant changes were observed in *Lactobacillus* or *Ruminococcus* (*p* > 0.05). Although *Bacteroides* abundance did not differ significantly between the treatment and D groups (*p* > 0.05), it was numerically lower in all treatment groups. *Bifidobacterium* abundance also showed an overall increasing trend following treatment and was numerically higher than that in the D group, although the differences were not statistically significant (*p* > 0.05).

Genus-level differences in the gut microbiota among groups were assessed based on community relative abundances. Compared with the D group, the C group was significantly enriched in *Lactobacillus*, *Bifidobacterium*, *Adlercreutzia*, and *Allobaculum*, whereas *Sutterella*, *Bacteroides*, *Paraprevotella*, and *Desulfovibrio* were significantly depleted (*p* < 0.05; Figure S2A). Relative to the D group, the L group was significantly enriched in *Turicibacter* and *Lactobacillus* (Figure S2B), while the M group was enriched in *Parabacteroides* and *Prevotella* (Figure S2C). The H group showed significant enrichment of *Akkermansia*, *Parabacteroides*, *Turicibacter*, *Bifidobacterium*, and *Allobaculum*, accompanied by significant depletion of *Oscillospira* and *Desulfovibrio* (*p* < 0.05, Figure S2D). These findings suggest that CD12-1 modulated the gut microbiota of mice with colitis by increasing potentially beneficial taxa, including *Lactobacillus*, *Prevotella*, and *Turicibacter*, while reducing inflammation-associated taxa such as *Desulfovibrio*.

### 3.6. Correlation Between Gut Microbiota and Colitis-Related Indicators

**Figure 6:**
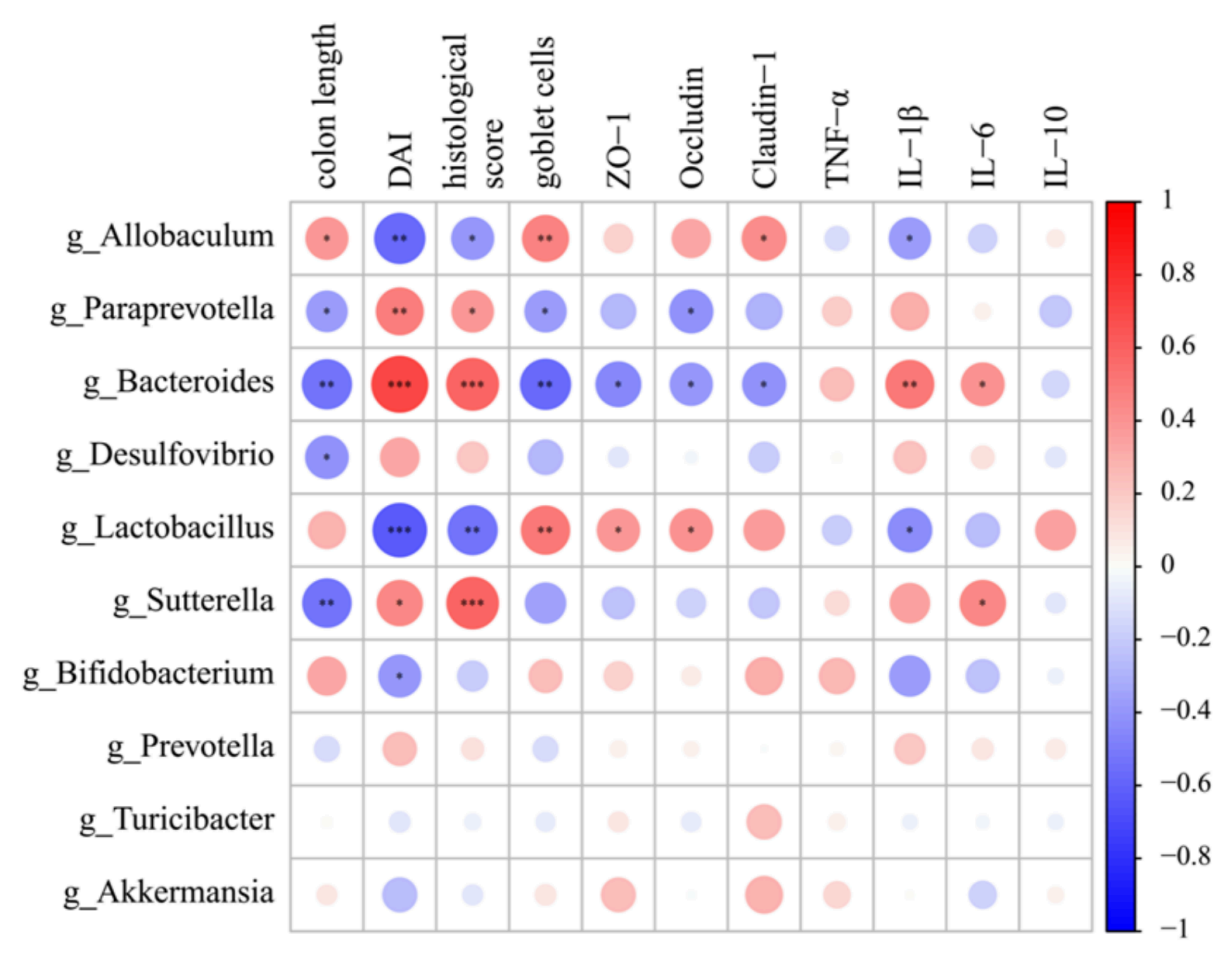
Correlation between gut microbiota and colitis-related indicators. n = 6 mice per group. * *p* < 0.05, ** *p* < 0.01, *** *p* < 0.001.

Correlation analysis revealed distinct associations between key bacterial genera and colitis-related parameters (Figure 6). *Lactobacillus* was negatively correlated with DAI, histological score, and IL-1β, but positively correlated with goblet cell number, ZO-1, and Occludin (*p* < 0.05). Similarly, *Allobaculum* was positively associated with colon length, goblet cell number, and Claudin-1, while being negatively associated with DAI, histological score, and IL-1β (*p* < 0.05). In contrast, *Bacteroides* exhibited the most pronounced adverse correlation pattern, showing positive correlations with DAI, histological score, IL-1β, and IL-6 and negative correlations with colon length, goblet cell number, ZO-1, Occludin, and Claudin-1 (*p* < 0.05). *Paraprevotella* was positively correlated with DAI and histological score but negatively correlated with colon length, goblet cell number, and Occludin, whereas *Sutterella* was positively correlated with DAI, histological score, and IL-6 and negatively correlated with colon length (*p* < 0.05). In addition, *Bifidobacterium* was negatively correlated with DAI, and *Desulfovibrio* was negatively correlated with colon length (*p* < 0.05). No significant correlations were detected for *Prevotella*, *Turicibacter*, or *Akkermansia*, and none of the examined genera were significantly associated with TNF-α or IL-10.

### 3.7. CD12-1 Increased the Concentration of SCFAs in the Gut

**Figure 7:**
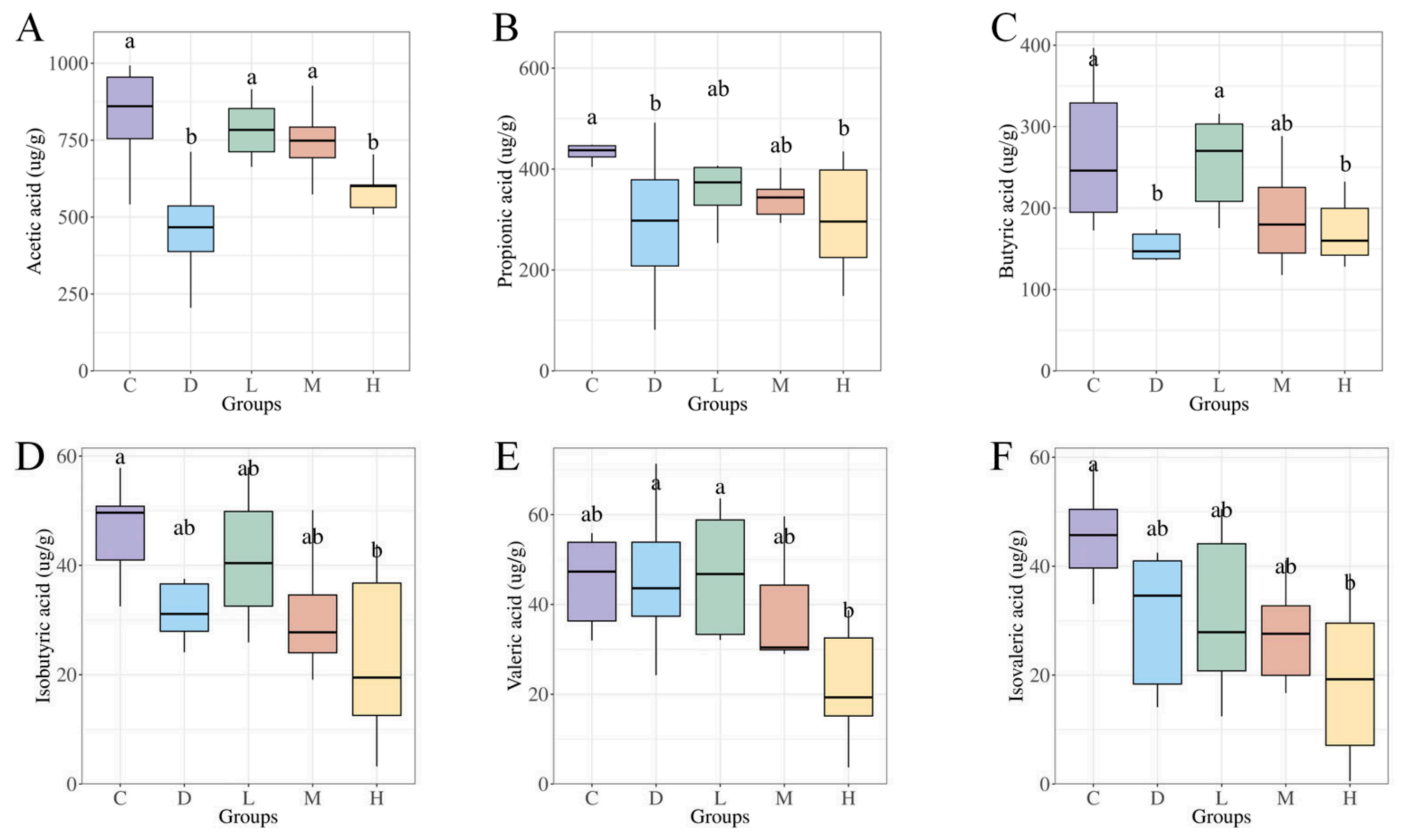
CD12-1 increased the concentration of SCFAs in the gut. (**A**) Acetic acid; (**B**) propionic acid; (**C**) butyric acid; (**D**) isobutyric acid; (**E**) valeric acid; (**F**) isovaleric acid. Data are presented as mean ± SEM (n = 6 mice per group). Different letters indicate significant differences among groups (*p* < 0.05).

As shown in Figure 7, the concentrations of acetic acid, propionic acid, and butyric acid were significantly lower in the D group than in the C group (*p* < 0.05), whereas no significant differences were observed in isobutyric acid, valeric acid, or isovaleric acid (*p* > 0.05). Compared with the D group, low-dose CD12-1 treatment significantly increased acetic acid and butyric acid concentrations in the L group, both of which were comparable to those in the C group (*p* > 0.05). Acetic acid concentration was also significantly higher in the M group than in the D group (*p* < 0.05) and did not differ significantly from that in the C group, whereas the other SCFAs remained unchanged (*p* > 0.05). In the H group, valeric acid concentration was significantly lower than that in the D group (*p* < 0.05), while no significant differences were observed for the other SCFAs (*p* > 0.05).

## 4. Discussion

DSS-induced mouse model of colitis reproduces several clinical and histopathological features of UC, including body weight loss, diarrhea, hematochezia, colon shortening, crypt damage, and inflammatory cell infiltration [16,17], and is therefore widely used to evaluate the effects of candidate probiotics on experimental colitis. In this study, treatment with 2.5% DSS resulted in body weight loss, an increased DAI, colon shortening, and marked histopathological injury, confirming the successful establishment of the experimental colitis model. Compared with the model group, intervention with CD12-1 generally attenuated body weight loss and clinical symptoms, reduced the DAI and histopathological damage, and partially alleviated colon shortening, indicating that this strain exerted a preventive protective effect against DSS-induced colitis.

Disruption of the intestinal mucosal barrier is a major pathological feature of DSS-induced colitis. Mucus secreted by goblet cells forms the first line of defense against luminal antigens and microbial invasion [18], whereas tight junction proteins, including ZO-1, Occludin, and Claudin-1, collectively maintain epithelial cell–cell adhesion and regulate intestinal permeability [19]. In this study, DSS treatment significantly reduced the number of colonic goblet cells and decreased the expression of ZO-1, Occludin, and Claudin-1, indicating impairment of both the mucus barrier and epithelial tight junctions. CD12-1 intervention markedly attenuated goblet cell loss and increased the expression levels of ZO-1 and Occludin, with the most pronounced improvements observed in the low-dose group. Although Claudin-1 expression showed an increasing trend following intervention, the between-group difference did not reach statistical significance (*p* > 0.05), suggesting that CD12-1 may exert differential regulatory effects on individual tight junction proteins. These findings are consistent with previous research results, indicating that CD12-1 may ameliorate DSS-induced mucosal barrier injury primarily by preserving the mucus layer and selected components of the epithelial tight junction complex [1,10].

Immune dysregulation serves a central role in the etiology of UC. Specifically, gut microorganisms and their metabolites translocate across the damaged intestinal barrier to the intestinal wall, successively activating innate and adaptive immunity, further causing increased production of pro-inflammatory cytokines and decreased production of anti-inflammatory cytokines, thereby resulting in a dysregulated immune response, which contributes to the initiation and progression of UC [1,2]. In this study, DSS treatment significantly increased the levels of IL-1β, IL-6, and TNF-α in colonic tissues while reducing the level of the anti-inflammatory cytokine IL-10, indicating disruption of the local balance between pro- and anti-inflammatory responses [20]. Low-dose CD12-1 intervention significantly decreased the levels of these pro-inflammatory cytokines and increased IL-10, suggesting that the strain alleviated DSS-induced local inflammation. These findings were consistent with the observed reductions in histopathological injury, restoration of goblet cells, and improvement in tight junction protein expression, indicating that suppression of inflammation and preservation of the mucosal barrier may jointly contribute to the protective effects of CD12-1 against colitis.

The composition and related abundance of the gut microbiome have a significant effect on the host immune system [21]. An imbalance of the intestinal microbiota is one of the key factors in the pathogenesis of UC [20,22]. Therefore, the use of probiotics to prevent or treat UC has great potential. The results of this study revealed that DSS exposure did not significantly alter the Chao1 or Shannon index relative to the C group, whereas CD12-1 exerted dose- and index-dependent effects on α-diversity. A similar endpoint-dependent response was observed in a previous study of *Lacticaseibacillus rhamnosus* G7, in which DSS did not significantly alter the Shannon or Simpson index relative to the control, whereas medium- and high-dose probiotic interventions significantly affected these indices [23]. Other studies have also reported a slight but non-significant increase in α-diversity following DSS exposure [24,25], suggesting that changes in within-sample microbial diversity depend on DSS concentration, exposure duration, sampling time, and the specific intervention applied [25,26].

Subsequent analysis of gut microbial composition showed that CD12-1 intervention restored the relative abundances of *Lactobacillus*, *Bifidobacterium*, *Allobaculum*, and *Akkermansia*, while also modulating those of *Bacteroides*, *Sutterella*, *Desulfovibrio*, and *Mucispirillum*. *Lactobacillus* possess probiotic potential and can produce bioactive compounds, including organic acids, bacteriocins, and exopolysaccharides [27]. These bacteria may contribute to intestinal homeostasis by inhibiting pathogens, preserving mucosal barrier integrity, and modulating host immune responses [11]. Similarly, *Bifidobacterium* has been reported to regulate gut microbial communities, suppress intestinal inflammation, and improve the mucosal environment [28,29]. The inflammatory milieu induced by DSS can promote abnormal shifts in inflammation-associated or potentially pathogenic taxa. The presence of *Bacteroides* can induce toxin production, which in turn causes intestinal inflammation in humans and animals, and *Bacteroides* produces succinic acid, which further exacerbates UC [30,31]. Therefore, a change in the abundance of *Bacteroides* may be an important indicator of UC. For *Desulfovibrio*, previous studies have reported increased relative abundance in the gut microbiota of mice with UC [1]. As sulfate-reducing bacteria, members of this genus can generate hydrogen sulfide; when local hydrogen sulfide production exceeds the oxidative detoxification capacity of the colonic epithelium, it may impair epithelial energy metabolism and compromise barrier function [32]. *Sutterella* is a genus of Gram-negative bacteria whose direct pro-inflammatory role remains unclear, although some studies suggest that it may influence mucosal immune regulation and treatment responses in patients with UC [33]. In addition, increased relative abundance of *Oscillospira* following DSS exposure has been reported in UC [34]. Correlation analysis further showed that *Lactobacillus* and *Allobaculum* were associated with lower DAI values, histopathological scores, and IL-1β levels, while being positively correlated with goblet cell abundance and the expression of selected tight junction proteins. In contrast, *Bacteroides* and *Sutterella* were primarily positively associated with disease severity and inflammatory indicators. These correlations indicate associations between specific microbial taxa and colitis-related phenotypes but do not establish that these taxa directly mediate the protective effects of CD12-1.

SCFAs are important metabolites produced by microbiota in the gut and contribute to the maintenance of epithelial energy metabolism, mucosal barrier integrity, and immune homeostasis [35,36,37]. Previous studies have reported that SCFA levels are commonly reduced in DSS-induced colitis, a change closely associated with gut microbial dysbiosis [10]. Probiotic supplementation may restore microbial metabolic function by reshaping the gut microbiota, thereby increasing SCFA production and alleviating intestinal barrier damage [11,38]. In this study, the effects of CD12-1 on SCFA levels varied among doses. The low-dose group showed marked increases in acetate and butyrate, whereas the medium-dose group mainly exhibited an increase in acetate, and the high-dose group did not show comparable improvements. Notably, gut microbiota analysis revealed an increased relative abundance of Ruminococcaceae in the CD12-1 treated groups. Although members of Ruminococcaceae include known butyrate-producing bacteria [39], the parallel changes in microbial composition and SCFA concentrations in this study do not directly demonstrate that the altered microbiota was responsible for the increased butyrate levels. Moreover, the present 16S rRNA gene analysis does not directly resolve microbial metabolic activity. Thus, the microbiota and SCFA findings should be interpreted as associated responses to CD12-1 intervention rather than as evidence of a causal microbiota–SCFA–barrier pathway.

Notably, the effects of CD12-1 did not follow a monotonic dose–response pattern. The high dose more effectively attenuated body weight loss, whereas the low dose produced significant improvements across a broader range of histological, barrier, inflammatory, and SCFA-related endpoints. Non-linear responses to probiotic administration have also been reported previously [40], indicating that higher probiotic doses do not necessarily result in greater host benefits. The biological basis of the response observed here remains unclear because intestinal abundance, persistence, and metabolic activity of CD12-1 were not directly quantified. More extensive dose-ranging studies combined with strain-specific tracking and functional analyses will be required to define the dose–response relationship and its underlying basis.

Despite these findings, several limitations should be considered. Because the present study was conducted exclusively in female C57BL/6 mice using an acute DSS-induced colitis model, the findings may not fully capture sex-related variability or the chronic, relapsing, and multifactorial nature of human UC. Moreover, because CD12-1 was administered before and during DSS exposure, the present data primarily support a preventive effect rather than therapeutic efficacy against established colitis. The gut microbiota analysis was based on six biological replicates per group, which may limit the statistical power to detect modest differences in microbial taxa; accordingly, microbiota-related findings should be interpreted as supportive associations and require validation in larger cohorts. Further validation in both sexes, complementary colitis models, and larger cohorts, followed by clinical studies, is warranted to establish the translational relevance of CD12-1.

## 5. Conclusions

In summary, fermented yak milk-derived CD12-1 alleviated DSS-induced colitis in mice by reducing clinical and histopathological injury, improving mucus and tight junction barriers, and modulating inflammatory responses, accompanied by changes in SCFA levels and gut microbial composition. The effects varied across doses, with the low-dose group showing the most comprehensive protection. These findings support the further development of CD12-1 as a candidate probiotic, although its key molecular mechanisms and optimal intervention dose require further validation.

## Figures and Tables

**Table 1:** DAI scoring criteria for colitis.

Score	Weight Loss (%)	Stool Consistency	Blood in Stool
0	0	Normal	No color development within 2 min
1	1–5	Loose stool without perianal adherence	Color gradually changes from light green to green after 10 s
2	5–10	Loose stool with perianal adherence	Immediate light-green color after reagent addition, gradually changing to blue-brown
3	10–15	Watery diarrhea	Immediate blue-brown color after reagent addition, gradually changing to dark brown
4	>15	Severe diarrhea	Immediate dark blue-brown color after reagent addition

## Data Availability

The original contributions presented in the study are included in the article/Supplementary Materials, further inquiries can be directed to the corresponding authors.

## Supplementary Materials

The following supporting information can be downloaded at: https://www.mdpi.com/article/10.3390/foods15173120/s1, Figure S1: Comparison of the relative abundances of different microbial taxa at the phyla (A) and genus (B) level; Figure S2: Genus-level differential abundance of the gut microbiota among groups. (A) C group vs. D group; (B) D group vs. L group; (C) D group vs. M group; (D) D group vs. H group.
