# Supplementary material for: Preventive Effects of Fermented Yak Milk-Derived Lacticaseibacillus paracasei CD12-1 Against DSS-Induced Colitis in Mice
Source: Foods. 2026 Sep 2;15(17):3120. doi: 10.3390/foods15173120 (PMC13564950; doi:10.3390/foods15173120)
Supplement: Supplementary file 1 [file foods-15-03120-s001.zip › foods-4514014-supplementary.pdf]

Supplementary Figures

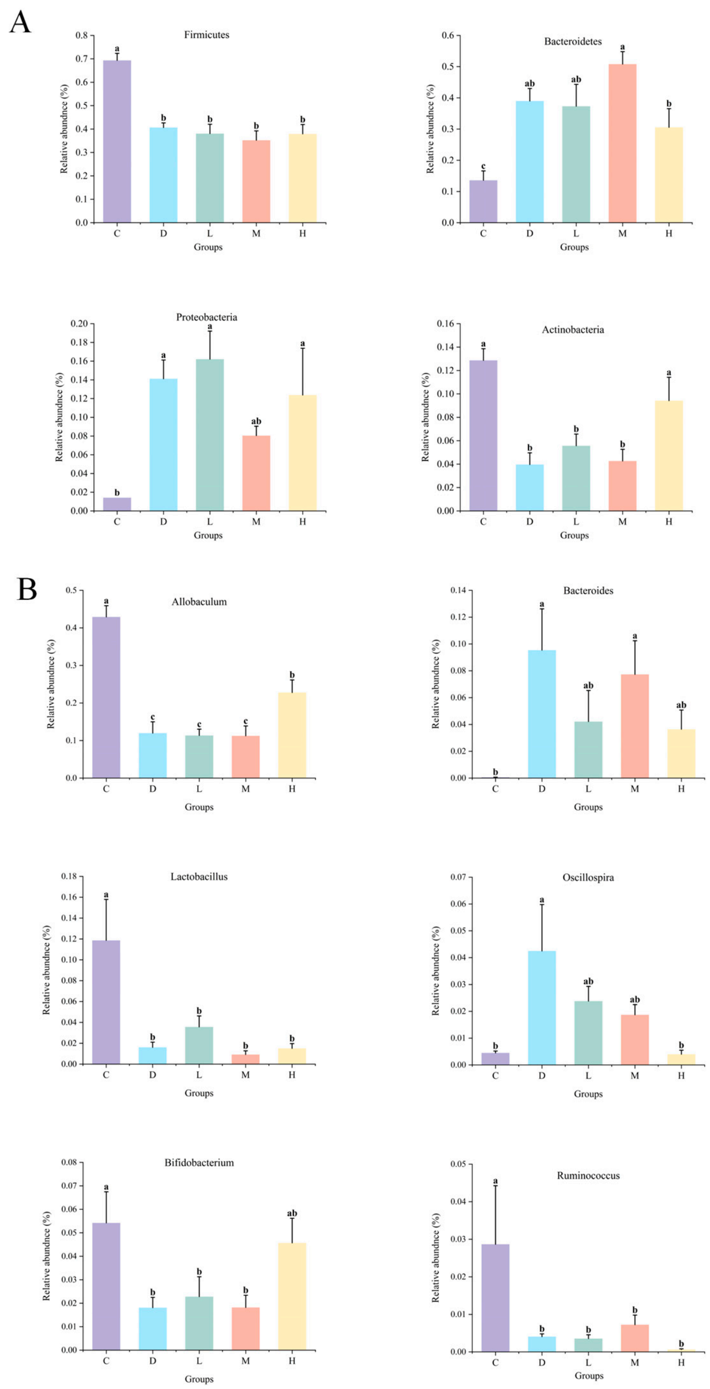

Figure S1. Comparison of the relative abundances of different microbial taxa at the phyla (A) and genus (B) level.

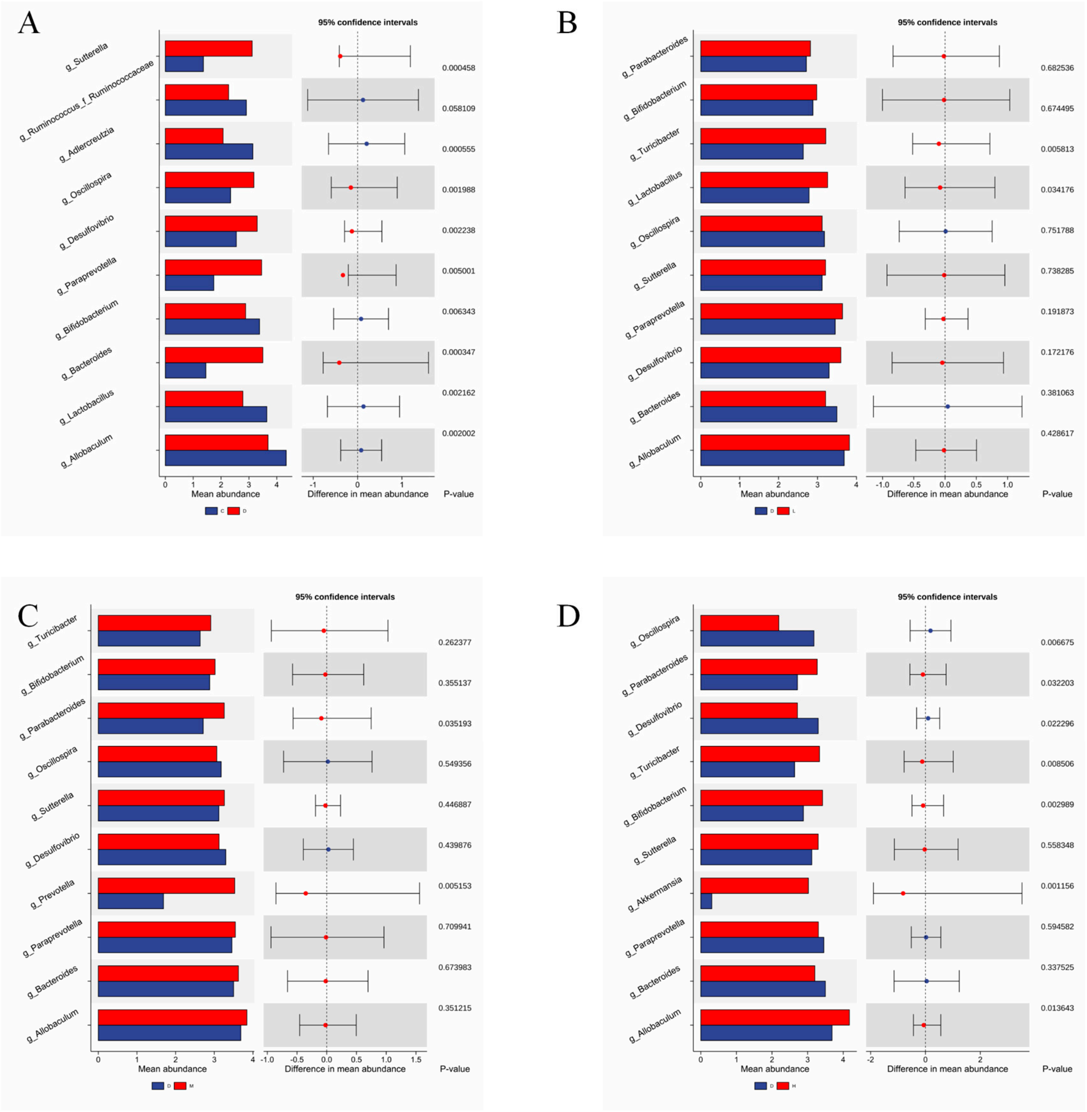

Figure S2. Genus-level differential abundance of the gut microbiota among groups. (A) C group VS D group; (B) D group VS L group; (C) D group VS M group; (D) D group VS H group.
